# Effective use of a supraglottic airway (i-gel™) during emergence from anesthesia in a patient with multiple giant bullae

**DOI:** 10.1186/s40981-024-00757-6

**Published:** 2024-11-28

**Authors:** Hayato Arime, Takashi Asai, Asuka Fujishiro, Tomoyuki Saito

**Affiliations:** Department of Anesthesiology, Dokkyo Medical University Saitama Medical Center, 2-1-50, Minami-Koshigaya, Koshigaya, Saitama 343-8555 Japan

**Keywords:** Deep extubation, Giant bulla, Laryngeal mask airway

## Abstract

**Background:**

Anesthetic management of a patient with multiple giant bullae is generally difficult due to an increased risk of respiratory complications, and there is no consensus regarding safe extubation methods. We report a case of an effective use of a supraglottic airway (i-gel™) during emergence from anesthesia in a patient with multiple giant bullae, in whom a double-lumen bronchial tube was being used during anesthesia.

**Case presentation:**

A 52-year-old man with multiple giant bullae underwent video-assisted pulmonary resections, while the ventilation was controlled via a double-lumen bronchial tube. After successful thoracoscopic surgery, an i-gel™ was inserted while the double-lumen tube was still in place, and the double-lumen tube was subsequently removed under deep anesthesia. The i-gel™ was removed without complications after the patient had become able to respond to verbal command.

**Conclusion:**

We believe that this method would minimize the risk of trauma to the respiratory system during emergence from anesthesia in patients with multiple giant bullae.

## Background

Tracheal extubation after general anesthesia may frequently be associated with respiratory complications, such as straining (bucking), breath-holding, coughing, laryngospasm, and bronchospasm [1–3]. A double-lumen bronchial tube, which is used during thoracic surgery to achieve one-lung ventilation, has a wide diameter and more rigid tubes than a single-lumen bronchial tube, and thus extubation of a double-lumen tube is associated with a higher incidence and great degree of respiratory complications [4].

Tracheal extubation when performed under deep anesthesia reduces the incidence of coughing, bucking, and the hemodynamic changes, but may increase the incidence of upper airway obstruction [2, 5]. This latter complication can be minimized by inserting a supraglottic airway, such as the laryngeal mask airway or the i-gel^TM^, under deep anesthesia [6]. A supraglottic airway may be inserted after tracheal extubation [7], but a better method is inserting a supraglottic airway *before* tracheal extubation [5, 6]. There have been no reports of the insertion of a supraglottic airway before the removal of a double-lumen tube during emergence of anesthesia to minimize respiratory complication.

In this report, we describe a case of an effective use of a supraglottic airway (i-gel™) during emergence from anesthesia in a patient with multiple giant bullae in whom a double-lumen bronchial tube was being used during anesthesia.

## Case presentation

A written consent was obtained from the patient for reporting this case and images.

**Fig. 1:**
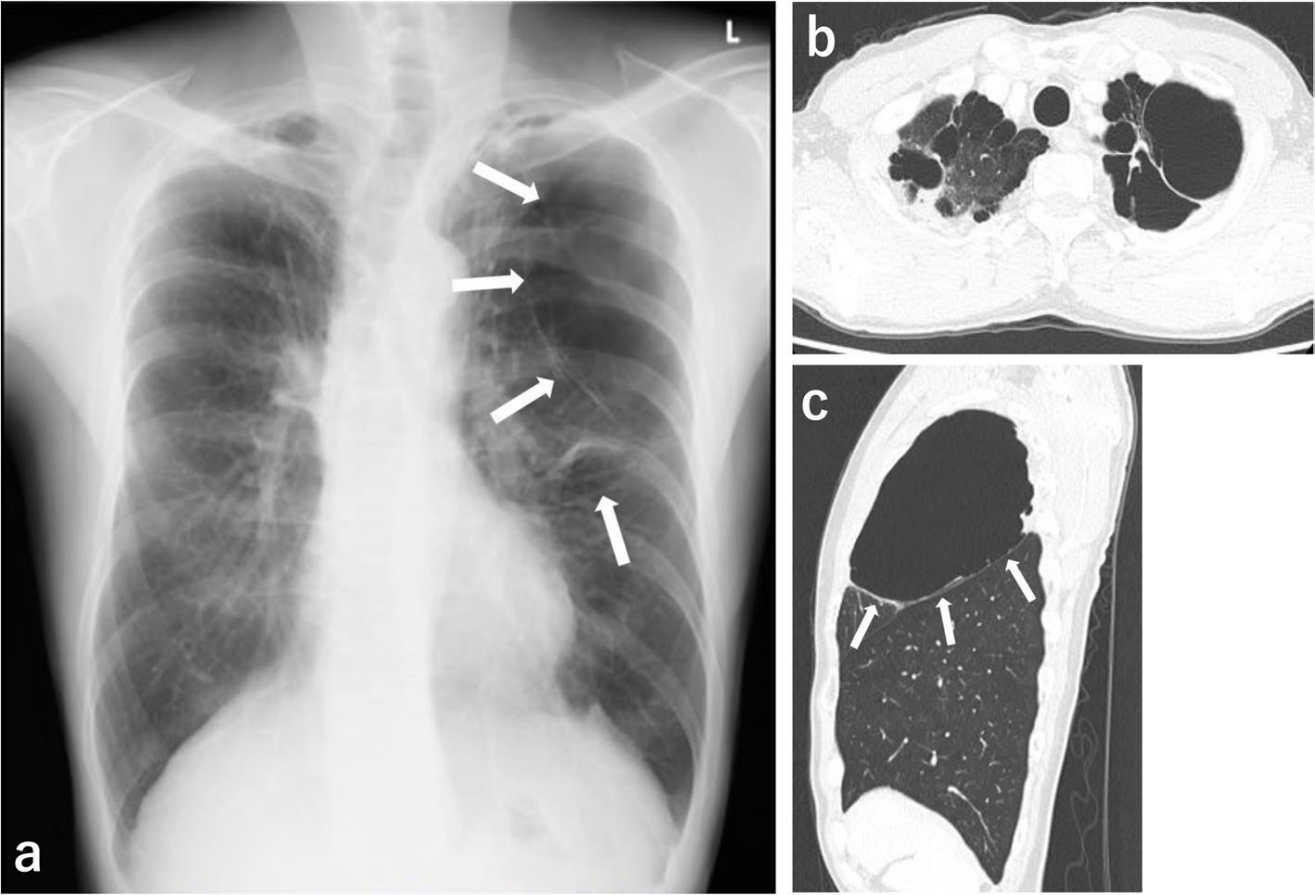
Pre-operative chest radiography (**a**) showing bulla on the left side (*arrows*). Horizontal slice (**b**) and sagittal slice (**c**) of computed tomography showing bilateral diffuse emphysematous changes, multiple bullae in the right upper lobe, and a giant bulla, in the left upper lobe (*arrows*)

A 52-year-old man (173 cm, 58 kg, BMI 19 kg m^−2^) with multiple giant bullae was scheduled for video-assisted pulmonary resections, because of repeated infection of the cysts. Preoperatively, chest radiography and computed tomography indicated a giant bulla in the left upper lobe, occupying the one-third of the left thoracic cavity, multiple bullae in the right upper lobe, and diffuse emphysematous changes of both lungs (Fig. 1). Respiratory function tests were within the normal limits, although the patient had shortness of breath during walking. He underwent resection of a right-sided giant emphysema 5 years ago.

On arrival at the operating room, routine monitors, such as a non-invasive blood pressure cuff, an electrocardiogram, a pulse oximeter, and bispectral index monitor, were applied. Before induction of anesthesia, the blood pressure was 147 / 99 mmHg, heart rate 80 beats·min^−1^, and oxygen saturation 99% on room air.

**Fig. 2:**
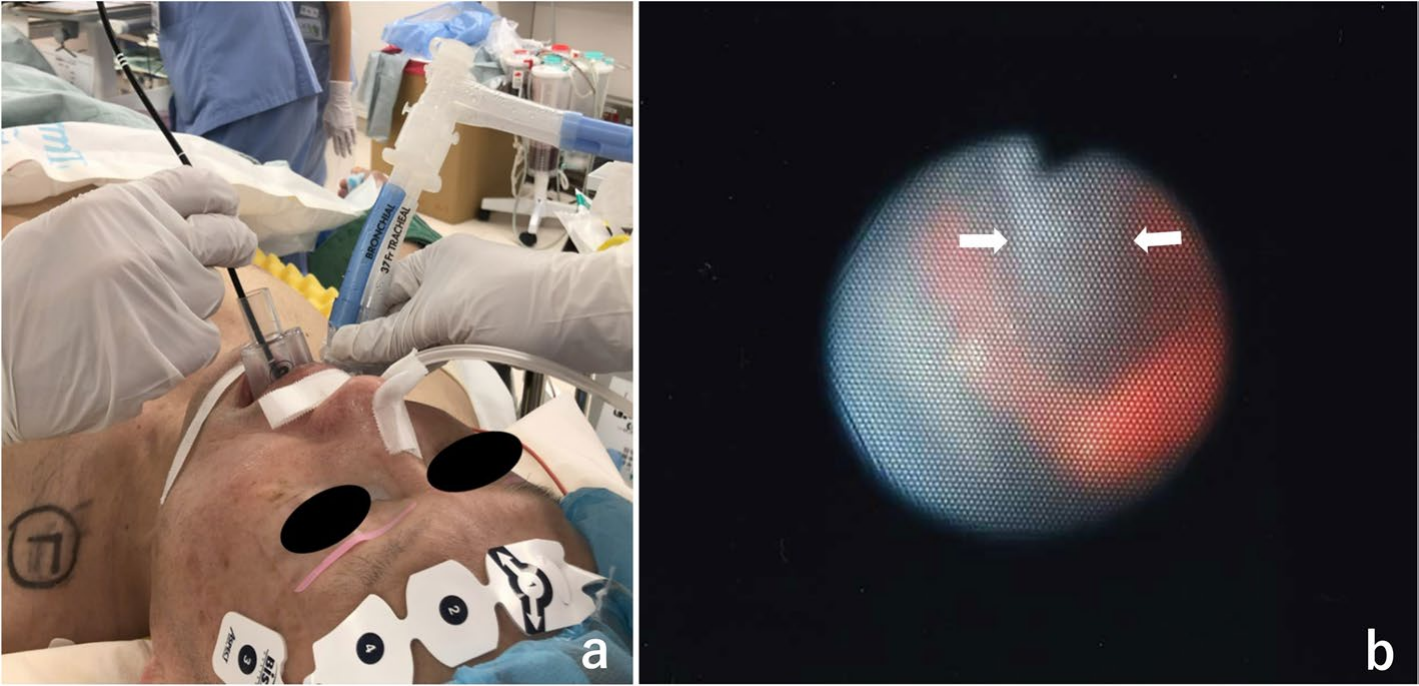
A fiberoptic bronchoscopy passing through an i-gel™, which was inserted while a double-lumen tube is still in place in an anesthetized patient, the size 3 i-gel™ inserted behind a double-lumen bronchial tube **a**. The view of a fiberscope which was inserted to the i-gel™ **b**. The double-lumen tube (*arrows*) is being inserted between the vocal cords in the center of the screen, indicating that the i-gel™ is inserted to the correct position

After preoxygenation of the patient via a face mask for more than 3 min, anesthesia was induced with propofol at target concentrations of 3 µg·ml^−1^ using a target-controlled infusion pump (TE-371, Terumo, Tokyo, Japan), fentanyl 100 µg, remifentanil at 0.25 µg·kg^−1^·min^−1^, and rocuronium 50 mg. Mask ventilation was easy. After confirmation of neuromuscular blockade, a 37-Fr left-side double-lumen bronchial tube (Shiley^TM^ endobronchial tube, Covidien Japan, Tokyo, Japan) was inserted with some difficulty, mainly due to limited mouth opening (Fig. 2). The patient was turned to the right decubitus position, and one-lung ventilation was started with the pressure-controlled ventilation, with the peak pressure of 14 cmH_2_O, PEEP 5 cmH_2_O (generating tidal volumes from 280 to 320 mL), inspiratory to expiratory (I:E) ratio: 1:1.5, and a fraction of inspired oxygen (FiO2) between 0.6 and 0.75. Anesthesia was maintained with continuous administration of propofol in the range of target concentrations of 2.5–2.7 µg·ml^−1^, remifentanil 0.15–0.25 µg·kg^−1^·min^−1^, and intermittent administration of fentanyl and rocuronium. Thoracoscopic left upper lobectomy and left pulmorrhaphy were performed without notable events.

Considering the possibility of air leakage from emphysematous lung tissues around the sutured areas and the fragility of the remaining lung tissues, the thoracic surgeons requested us to avoid increased airway pressure such as by straining or coughing as much as possible during emergence of anesthesia.

Under deep anesthesia, a size 3 i-gel™ was easily inserted orally while the doublelumen bronchial tube was still in place. A fiberoptic bronchoscope was inserted into the breathing tube of the i-gel™ (Fig. 2a) to confirm the position. It was possible to observe, beyond the distal aperture of the i-gel™ that, the bronchial tube was passing through the glottis, indicating that the i-gel™ was placed in the correct position (Fig. 2b). The cuffs of the double-lumen bronchial tube were deflated, and the bronchial tube was removed while the i-gel™ was kept in place. The breathing system was detached from the bronchial tube and connected to the i-gel™, and adequacy of ventilation through the i-gel™ was confirmed by adequate chest expansion, sufficient tidal volumes and the presence of end-tidal carbon dioxide waveforms. Neither straining nor coughing occurred.

Continuous infusion of propofol and remifentanil was then stopped, and neuromuscular blockade was antagonized by sugammadex 200 mg. The patient started to breathe spontaneously, and became able to respond to the verbal command, and thus the igel was removed. No respiratory complications, such as coughing and bucking, were observed. Postoperative arterial blood gas analysis was within the normal limits, and chest radiographs indicated no rupture of remaining bullae or pneumothorax. The postoperative course was uneventful, and the patient was discharged from our hospital on postoperative day 7.

## Discussion

Pulmonary bulla is defined as pathologically dilated air sac of more than 2 cm diameter and when the bulla occupies more than one-third or the half of the lung volume is considered giant [8]. Anesthetic management of patients with giant bulla is generally difficult due to the risk of respiratory complications, such as pneumothorax and atelectasis. In particular, a giant bulla or multiple bullae remained present after surgery; there would be an increased risk of trauma to the respiratory system during emergence from anesthesia, because the presence of the bronchial tube may frequently cause straining or coughing.

By inserting a supraglottic airway while the double-lumen tube was in place and subsequently removing the double-lumen tube under deep anesthesia, and by maintaining a clear airway with the supraglottic airway until the patient could respond to verbal command, we observed no straining or coughing during emergence from anesthesia and could avoid trauma to the respiratory system. Pharmacological agents such as lidocaine, fentanyl, remifentanil, and dexmedetomidine may be effective in facilitating smooth extubation [8–11] using these drugs in addition to the replacement of the double-lumen tube to a supraglottic airway may be more effective. We did not use these drugs during extubation in this case, because the patient was still deeply anesthetized.

A possible problem with this technique is that, if the supraglottic airway is not inserted to the correct position, airway obstruction may occur after the tracheal extubation. A supraglottic airway may frequently be placed in a suboptimal position, particularly when performed by inexperienced personnel [12–14]. Therefore, it is advisable to use a fiberoptic bronchoscope to confirm that the supraglottic airway is placed to the optimal position, before removal of the bronchial tube.

We conclude that, in a patient in whom a double-lumen bronchial tube was being inserted during anesthesia, insertion of a supraglottic airway while the double-lumen tube is in place and subsequent removal of the double-lumen tube under deep anesthesia, would minimize the risk of trauma to the respiratory system during emergence from anesthesia.

## Data Availability

The data in this case report are available from the corresponding author on reasonable requests.
