# Correction: Effective use of a supraglottic airway (i-gel™) during emergence from anesthesia in a patient with multiple giant bullae

**DOI:** 10.1186/s40981-025-00768-x

**Published:** 2025-01-22

**Authors:** Hayato Arime, Takashi Asai, Asuka Fujishiro, Tomoyuki Saito

**Affiliations:** Department of Anesthesiology, Dokkyo Medical University Saitama Medical Center, 2‑1‑50, Minami‑Koshigaya, Koshigaya, Saitama 343‑8555 Japan

**Correction: JA Clin Rep 10, 73 (2024)**

**https://doi.org/10.1186/s40981-024-00757-6**

Following publication of the original article [1], the author reported that two paragraphs just before "Discussion" section were removed during production process. Please see below for the missing paragraph.

“Under deep anesthesia, a size 3 i-gelTM was easily inserted orally while the doublelumen bronchial tube was still in place. A fiberoptic bronchoscope was inserted into the breathing tube of the i-gelTM (Fig. 2a) to confirm the position. It was possible to observe, beyond the distal aperture of the i-gelTM that, the bronchial tube was passing through the glottis, indicating that the i-gelTM was placed in the correct position (Fig. 2b). The cuffs of the double-lumen bronchial tube were deflated, and the bronchial tube was removed while the i-gelTM was kept in place. The breathing system was detached from the bronchial tube and connected to the i-gelTM, and adequacy of ventilation through the i-gelTM was confirmed by adequate chest expansion, sufficient tidal volumes and the presence of end-tidal carbon dioxide waveforms. Neither straining nor coughing occurred.

Continuous infusion of propofol and remifentanil was then stopped, and neuromuscular blockade was antagonized by sugammadex 200 mg. The patient started to breathe spontaneously, and became able to respond to the verbal command, and thus the igel was removed. No respiratory complications, such as coughing and bucking, were observed. Postoperative arterial blood gas analysis was within the normal limits, and chest radiographs indicated no rupture of remaining bullae or pneumothorax. The postoperative course was uneventful, and the patient was discharged from our hospital on postoperative day 7.”

The original article has been updated to include the missing paragraphs.
